# Prevalence of subjective cognitive decline and its association with physical health problems among urban community dwelling elderly population in South India

**DOI:** 10.1002/alz.14505

**Published:** 2025-02-12

**Authors:** Jayakumar Menon, Suvarna Jyothi Kantipudi, Stanley Vinoth, Jyothsna Devi Kuchipudi

**Affiliations:** ^1^ Department of Psychiatry SRMC &RI Sri Ramachandra Institute of Higher Education and Research (SRIHER) Chennai India; ^2^ Department of Nutrition Sri Ramachandra Institute of Higher Education and Research (SRIHER) Chennai India; ^3^ School of Public Health University of California Berkeley California USA

**Keywords:** Elderly population, Montreal Cognitive Assessment (MoCA), Preclinical Alzheimer's Disease (PCAD), Subjective Cognitive Decline (SCD), Subjective Memory Complaints Questionnaire (SMCQ)

## Abstract

**INTRODUCTION:**

No studies in India have explored subjective cognitive decline (SCD), a hallmark of stage II of preclinical Alzheimer's disease. This study aims to assess the prevalence and correlates of SCD in a South Indian, urban, elderly population.

**METHODS:**

We screened 403 individuals 60 years of age and older using the Subjective Memory Complaints Questionnaire (SMCQ) and measured objective cognition with the Montreal Cognitive Assessment (MoCA). Physical health parameters were evaluated for all participants.

**RESULTS:**

Among the participants, 377 (93.5%) reported subjective memory complaints. Of the 26 individuals without SCD, 15(57.7%) had objective cognitive impairment (MoCA <25). A total of 182 participants (45.2%) were identified with SCD. Higher educational attainment was significantly associated with fewer SCD reports and better cognitive performance (p < 0.001).

**DISCUSSION:**

Subjective cognitive decline (SCD) is highly prevalent among older adults. Screening for SCD can help identify individuals at risk for Alzheimer's disease. SCD assessement combined with cost‐effective biomarkers that confirms AD will help individuals to be identified for disease‐modifying therapies.

**Highlights:**

- Nearly half of older adults population screened has reported subjective cognitive decline (SCD), highlighting the widespread occurrence of SCD in urban South India.
- Participants with higher educational attainment had significantly fewer memory complaints and performed better on cognitive assessments.
- SCD was prevalent even among individuals without major comorbid conditions such as diabetes and hypertension and those who were on regular treatment for metabolic and cardiovascular disorders.
- Identifying subjective cognitive decline (SCD) can facilitate early and accurate diagnosis of cognitive disorders and help delay progression to dementia. This highlights the importance of developing and implementing improved public health strategies to address these challenges.
- Further longitudinal studies are necessary to explore the progression of SCD to dementia, focusing on the interplay between cognitive health, biomarkers, and educational factors in the Indian population.

## 1 BACKGROUND

Cognitive disorders and dementia are leading causes of morbidity and early mortality globally.^1^, ^2^ Their prevalence is increasing, particularly in low‐ and middle‐income countries.^3^ Among various etiologies, Alzheimer's disease (AD) and related neurodegenerative disorders remain the most common irreversible causes.India has a burgeoning elderly population, projected to reach 132 million individuals 60 years of age and older by 2026 (UNPF, HelpAge India).^4^ Consequently, the number of dementia cases is expected to rise exponentially over the next two decades. Unfortunately, there is a huge gap in dementia care–related services in India and most of them remain concentrated in major metropolitan cities. There are no recent prevalence studies on cognitive disorders from the country except the Longitudinal Aging Study in India (LASI), which has provided an estimated prevalence.^5^

The progression of AD spans pre‐clinical, mild cognitive impairment (MCI), and mild, moderate, and severe stages of dementia.^6^ Identifying illness at the earliest stage has become a critical focus in recent years, as disease‐modifying agents are most effective in earlier stages.^7^ Subjective cognitive decline (SCD), a key feature of preclinical AD (PCAD) involves self‐reported cognitive difficulties, without evidence of objective cognitive impairment or functional decline. Notably, 50% of individuals who are in Stage II PCAD with SCD, progress to Alzheimer's dementia, highlighting its clinical significance.^8^SCD is typically identified through self‐reports and quantified using specific scales. There have been efforts to develop a consensus on conceptualizing and assessing SCD.^9^ Studies have also explored categorizing SCD with biomarker assays, to predict the risk of dementia.^10^ With the increasing availability of blood biomarkers and disease‐modifying therapies (DMTs), accurately identifying individuals at risk of progression to Alzheimer's dementia is important.

Community screening for SCD coupled with cost‐effective blood biomarkers may be pivotal in early identification and mitigating the impending dementia epidemic in low‐ and middle‐income countries (LMICs). Studies in high‐income countries have looked at the progression of SCD in elderly population, but the findings have been inconsistent.^11^, ^12^ A recent meta‐analysis, however, indicated that individuals with SCD and AD pathology are at much higher risk for developing dementia than those without AD pathology.^13^However, there are no studies from India examining the prevalence of SCD. This study aimed to determine the prevalence of SCD in a community‐dwelling population 60 years of age and older, and its association with various physical health parameters.

## 2 METHODS

### 2.1 Study design

This was a cross‐sectional observational study conducted among individuals 60 years of age or older. The study design and data description adhered to the principles of observational studies, following the Strengthening the Reporting of Observational Studies in Epidemiology (STROBE) guidelines.

### 2.2 Setting

This study was conducted by the Geriatric Care Clinic at a tertiary teaching hospital, Sri Ramachandra Institute of Higher Education and Research (SRIHER), located in the suburbs of Chennai, the capital city of the state of Tamil Nadu in South India. It boasts a well‐established health and social support infrastructure but faces significant disparities in income, social indices, and health care–seeking behaviors.

An increasing number of elderly individuals in urban society are left to fend for themselves due to various factors, including the migration of children overseas, the shift from joint or extended family systems to nuclear families, and longer life expectancy. These trends heighten the risk of social isolation and contribute to a rise in physical and psychological disorders among older adults.

*Participants*: The study included community‐dwelling elderly individuals 60 years of age or older from both genders, selected from various settings. These settings included independent homes near the hospital, elderly care homes, individuals visiting the center for regular health check‐ups, and participants from community health camps. This diverse selection ensured a representative sample of older adult population and minimized selection bias.

**RESEARCH IN CONTEXT**

1. **Systematic review**: Previous studies focused on cognitive disorders such as Alzheimer's disease (AD), mild cognitive impairment (MCI), and subjective cognitive decline (SCD) have come primarily from developed countries. There is limited research on SCD in India, particularly in community‐dwelling elderly populations. Our study addresses this gap by evaluating the prevalence of SCD in an urban South Indian setting, contributing new insights to global dementia research.
2. **Interpretation**: Our findings show a high prevalence of SCD in older adults population of South India, with nearly half experiencing pre‐MCI SCD. Education emerged as a significant protective factor, with individuals having higher educational attainment showing better cognitive function and less SCD. These results emphasize the importance of early screening in identifying at‐risk individuals for dementia, and the role of education in mitigating the progression of cognitive disorder in low‐ and middle‐income countries.
3. **Future directions**: Further longitudinal studies are needed to explore the usefulness of SCD screening in the community in the Indian context, with a focus on integrating cost‐effective biomarkers (blood biomarkers) for early and accurate diagnosis of AD and other neurodegenerative disorders. In addition, expanding community‐based screening programs and educational interventions could help prevent or delay the onset of dementia. Future research should aim to develop cost‐effective, scalable models for early cognitive screening and intervention in aging populations in India and other low‐resource settings.

The study was part of the comprehensive community‐based outreach program conducted by the Geriatric Care Clinic at SRIHER.

### 2.3 Inclusion and exclusion criteria

#### 2.3.1 Inclusion criteria

1. Individuals of any gender 60 years of age or older were eligible.
2. Participants who could provide informed consent to take part in the study.

#### 2.3.2 Exclusion criteria

1. Individuals with serious medical comorbidities that prevented participation in cognitive screening were excluded.
2. Participants with vision or hearing impairments that could interfere with evaluations were excluded.
3. Individuals diagnosed with neurodegenerative disorders were not eligible.
4. Individuals with current psychiatric disorders, particularly depression, were excluded.

### 2.4 Variables

Exposure variables included socio‐demographic details (age, gender, marital status, education, and socio‐economic status) and medical comorbidities (e.g., diabetes and hypertension), gathered through a semi‐structured proforma, family interviews, and medical records.

The primary outcome variables were:

1. Subjective Cognitive Decline(SCD) – assessed using the Subjective Memory Complaints Questionnaire (SMCQ).
2. Mild Cognitive Impairment(MCI) – evaluated using the Montreal Cognitive Assessment (MoCA).
3. Functional decline – measured with the Bristol Activities of Daily Living Scale (BADLS).

*Effect Modifiers*: Living situation and health‐seeking behaviour were hypothesized as potential effect modifiers but were not captured in this study.

All elderly individuals fulfilling the inclusion criteria and who could give informed consent were screened for the presence of comorbid medical and psychiatric illness using a combination of semi‐structured proforma and interviews, which also captured the socio‐demographic details. These included age, gender, marital status, education, socio‐economic status, and history of medical and psychiatric comorbidities. In addition, medical records were reviewed and interviews with the family members/staff nurses were done to confirm the details collected.

The primary author (J.M.), a senior consultant at the Geriatric Care Clinic of the hospital, has extensive experience in assessing and managing neurocognitive disorders, and is proficient in administering cognitive screening and neuropsychological assessment tools. The co‐author (S.J.K.), also a senior consultant at the same tertiary teaching hospital, contributed expertise in conceptualization and analysis of the study.

Other members of the research team (S.V., J.V.) are actively involved with the Geriatric Care Clinic and are skilled in patient assessment. Before conducting evaluations, each team member completed at least 15 supervised assessments to ensure proficiency. Their competence in administering cognitive screening tools was certified, and inter‐rater reliability was established. The primary author independently verified each case to check for potential errors in assessment and confirm the clinical diagnosis. The tools used for subject evaluation by the trained researchers are detailed below.

1. SMCQ: SMCQ is a 14‐item self‐reporting instrument for older adults that assesses memory problems in general and daily living.^14^ It has good psychometric properties and is used widely to assess subjective decline in cognition.^15^ The maximum score is 14, and a higher score indicates more severe SCD. For this study, even a score of 1 was considered indicative of SCD. The scale in English was used for individuals who were literate and fluent in English, whereas the translated and validated Tamil version of the scale was used for individuals fluent in Tamil only. For those who are illiterate, the questions were read out and their answers marked.
2. MoCA: MoCA is a 30‐item scale, with validated Tamil and Hindi versions, used extensively in memory clinics in India. Normative scores of the population are available. The cutoff score is 25; MCI is diagnosed when a person has impairment more than 1.5 standard deviation (SD) in any one or more cognitive domains without impairment in day to day activities, with a Clinical Dementia Rating (CDR) scale score of 0.5 or less.^15^ For our study, patients were classified into normal cognition (NC) (MoCA score 25–30) without SCD, NC with SCD, and cognitive impairment (MoCA score <25). This categorization provided the best possible distinction of patients, as those with MoCA scores in the normal category and no SCD were considered normal, except when there was a disproportionately greater impairment in any cognitive function more than 1.5 SD from the normal. This was arrived at with further clinical history and details of functional ability. (For example, Verbal recall being 3 or less than 3/5 was categorized as amnestic MCI, even though the total score was within normal range.
3. BADLS: The BADLS is a 20‐item informant‐rated scale used to evaluate the ability of individuals with cognitive impairment to perform activities of daily living.^16^ All participants assessed using the SMCQ and objective cognitive screening tools were also evaluated with the BADLS to identify functional decline. Participants were considered to have functional decline if they showed impairment in more than two items on the BADLS in conjunction with poor performance on cognitive screening tests. In addition, a cutoff score of 5 was used to distinguish participants without functional decline. This categorization was further validated through independent reports from residential center staff or family members. This approach was designed to clearly differentiate participants with normal cognitive function (with or without SCD) from those with objective evidence of cognitive impairment.

*Bias*: Many strategies were employed to eliminate potential bias in this study. Selection bias was minimized by recruiting participants from diverse settings, ensuring adequate representation of the sample. Information bias was reduced through data collection by trained researchers using standardized assessments tools.

*Study sample size*: There were no prevalence estimates of SCD from India. Assuming a 50% prevalence of SCD and conservatively estimating 1 million age 60 plus individuals in the city (10% of the population), the sample size needed for 95% confidence level (CI) was 385. A total of 418 individuals 60 years or older were found suitable for the study after screening.

*Statistical Methods*: The distribution of sociodemographic and clinical variables across different cognitive levels was evaluated using descriptive statistics (mean ± SD, frequency distributions). To evaluate continuous and categorical variables across the groups, inferential statistics including parametric tests (analysis of variance [ANOVA] and chi‐square tests) were used. Data were analyzed using SPSS (Statistical Package for the Social Sciences)Version 27.0.^17^

### 2.5 Statistical analysis

Quantitative variables in the study included age and scores from cognitive assessment tools. Age was treated as a continuous variable and presented as mean ± SD across three groups: NC, SCD, and cognitive impairment.

Cognitive assessment scores, including those from the Subjective Memory Complaints Questionnaire (SMCQ) and its component scores, were reported as mean ± SD for each group, highlighting differences in general functioning, everyday memory functioning, total scores, and delayed memory recall among the groups. Statistical analyses such as one way ANOVA were used to compare these variables across different cognitive levels.

## 3 RESULTS

### 3.1 Participants

A total of 418 individuals were examined for eligibility; of them, 403 met the eligibility criteria and were included in the study. Fifteen participants were excluded due to underlying psychiatric comorbidities. There were no missing data for the 403 participants included, and all of them were included in the analysis.

### 3.2 Descriptive data

The study included 403 urban community‐dwelling elderly individuals from South India, categorized into three cognitive status groups:

1. NC: MoCA score of 25–30 with no SCD (*N* = 11).
2. SCD: MoCA score of 25–30 with SCD (*N* = 182).
3. CI(Cognitive impairment): MoCA score <25, with or without SCD (*N* = 210).

The mean age across these groups was comparable: 65.64 years for the NC group, 68.9 years for the SCD group, and 69.14 years for the cognitive impairment group.

Gender distribution showed a slight female preponderance, with women comprising 53.3% of the participants. Education levels varied significantly across groups. Higher secondary school completion was more common in the NC group, whereas non‐completion of higher secondary education was prevalent in the cognitive impairment group. Employment status was evenly distributed, with approximately half of the participants retired or not working. Body mass index (BMI) categories and the prevalence of chronic conditions such as diabetes mellitus and hypertension did not show significant differences among the groups. It is important to note that there were no missing data for any of the variables of interest across the 403 participants included in the study.

### 3.3 Outcome data

The prevalence of diabetes mellitus was 37.5%, hypertension was 34.7%, and SCD was 45.2% in the urban elderly population studied.

### 3.4 Main results

The mean age across the three groups—NC, SCD, and cognitive impairment—was similar, with a slight increase in mean age in the cognitive impairment group. However, this difference was not statistically significant (*p* = 0.28). Gender distribution was also comparable across groups, with no significant difference (*p* = 0.99). The majority of participants in all groups were female.

A significant association was observed between education level and cognitive status (*p* < 0.001). Participants in the NC group were more likely to have completed higher secondary school, whereas those in the cognitive impairment group were more commonly non‐completers of higher secondary education. Employment status did not differ significantly among the groups (*p* = 0.99), with most participants in all groups having been employed.

SCD was more prevalent in participants with a BMI less than 18.5, whereas cognitive impairment and SCD were evenly distributed among participants with a BMI greater than 25. However, the overall difference was not statistically significant (*p* = 0.09). Similarly, the prevalence of diabetes mellitus and hypertension did not vary significantly across groups.

Functional assessment using the BADLS revealed that only one participant had a score greater than 5, indicating significant functional impairment. The remaining 402 participants scored between 0 and 5. The details are presented in Table 1.

**TABLE 1:** Demographic and physical health characteristics of an urban community dwelling elderly population in South India.

	NC (MoCA 25–30 No SCD) *N* = 11	SCD (MoCA 25–30 with SCD) *N* = 182	CI (MoCA <25) *N* = 210	*p*‐value
Age, years, mean ± SD	65.64 ± 7.12	68.9 ± 6.84	69.14 ± 7.0	0.28
**Gender, (*n* (%)**				
Male (188)	5 (2.7)	85 (45.2)	98 (52.1)	0.99
Female (215)	6 (2.8)	97 (45.1)	112 (52.1)	
**Education‐self, *n* (%)**				
Higher secondary school complete (266)	1 (0.4)	142 (53.4)	123 (46.2)	<0.001**
Higher secondary school incomplete (137)	10 (7.3)	40 (29.2)	87 (63.5)	
**Working status, *n* (%)**				
Employed (214)	6 (2.8)	96 (44.9)	112 (52.3)	0.99
Retired/not working (189)	5 (2.6)	86 (45.5)	98 (51.9)	
**BMI, *n* (%)**				
<18.5 (8)	1 (12.5)	5 (62.5)	2 (25)	0.05
18.5–22.99 (89)	4 (4.5)	44 (49.4)	41 (46.1)	
23–24.99 (78)	3 (3.8)	28 (35.9)	47 (60.3)	
>25 (228)	3 (1.3)	105 (46.1)	120 (52.6)	
**Diabetes mellitus, *n* (%)**				
Absent (252)	8 (3.2)	115 (45.6)	129 (51.2)	0.75
Present (151)	3 (2)	67 (44.4)	81 (53.6)	
**Hypertension, *n* (%)**				
Absent (263)	7 (2.7)	113 (43)	143 (54.4)	0.43
Present (150)	4 (2.9)	69 (49.3)	67 (47.9)	
**Functional assessment (BADLS score)**				
0–5 (402)	11 (2.7)	182 (45.3)	209 (52)	1.00
>5 (1)	0 (0)	0 (0)	1 (100)	

Abbreviations: BADLS, Bristol Activities of Daily Living Scale; BMI, body mass index; MoCA, Montreal Cognitive Assessment; SCD, subjective cognitive decline.

** Suggests *p* value less than 0.001

The presence of subjective cognitive complaints, general and everyday memory functioning scores, total SMCQ scores, and delayed memory recall scores were compared across the three cognitive groups. There were significant differences across groups with better scores in the NC group (*p* < 0.001).A strong association was found between subjective cognitive complaints and cognitive status (*p* < 0.001). Participants in the NC group predominantly reported no SCD, whereas SCD was highly prevalent in the cognitive impairment group. Participants in cognitive impairment group reported markedly lower scores in general and everyday memory functioning on SMCQ scale compared to those with NC (*p* < 0.001). Total SMCQ scores were also significantly lower in the cognitive impairment group.

Delayed memory recall scores were significantly lower in both the SCD and cognitive impairment groups compared to the NC group (*p* < 0.001), highlighting a decline in memory function among individuals with subjective complaints and cognitive impairment.

The details of these results are presented in Table 2.

**TABLE 2:** Cognitive health among urban community dwelling elderly population in South India.

	NC (MoCA 25–30, No SCD) *N* = 11	SCD (MoCA 25–30 with SCD) N = 182	CI (MoCA < 25) N = 210	*p*‐value
**Subjective memory complaint, *n* (%)**				
Absent	11 (42.3)	–	15 (57.7)	<0.001**
Present	**–**	182 (48.3)	195 (51.7)	
**SMCQ scores,** mean ± SD				
General functioning	–	3.44 ± 0.96	2.79 ± 1.35	<0.001**
Everyday memory functioning	–	8.05 ± 2.48	6.65 ± 3.01	
Total scores	–	11.49 ± 3.3	9.44 ± 4.22	
Memory scores, mean ± SD				
Delayed memory recall	4.09 ± 1.04	3.83 ± 0.73	2.43 ± 1.08	<0.001**

Abbreviations: MoCA, Montreal Cognitive Assessment; SMCQ, Subjective Memory Complaints Questionnaire.

** Suggests *p* value less than 0.001

## 4 DISCUSSION

To the best of our knowledge, this is the first study from India to assess the prevalence of subjective memory complaints in older adults population residing in the community. One of the major findings of our study was the high prevalence of SCD, which was observed in 45.16% of the population. This finding is not surprising, given the known high prevalence of dementia in the Indian population.^5^, ^18^ Close to 50% of the population with normal cognitive screening scores (above cutoff for MoCA, i.e., >25) had subjective memory complaints, which was higher than the prevalence observed in similar studies done in other parts of the world.^19^ In addition, a proportion of participants showed evidence of cognitive difficulties in objective tests, with about half of the participants exhibiting signs of possible cognitive impairment. This suggests a high prevalence of individuals at risk of progressing to MCI or dementia in the coming years, particularly if associated with AD or other dementia risks identified through biomarkers. Recent studies evaluating individuals with SCD using biomarkers have demonstrated that those with both SCD and positive biomarkers were at a significantly higher risk of progressing to MCI and dementia.^9^, ^12^

However, the findings from our community screening were concerning, as none of older adults participants had undergone cognitive screening, despite subjectively experiencing and likely reporting cognitive difficulties during multiple physician visits. This highlights a critical gap in awareness among general practitioners and specialists in the city regarding cognitive disorders. It also points to a lack of knowledge about the early signs of such disorders, which should prompt cognitive screening for individuals 60 years of age or older.

Considering the projected rise in dementia cases in India by 2030 and 2050,^5^ coupled with the anticipated demographic shift toward an older population,^4^ the findings from our study underscore an urgent need to improve awareness, enhance cognitive screening efforts for older adults, and advocate for increased resource allocation to establish services for cognitive disorder identification, management, and care. These efforts are essential to address the growing challenge of dementia in this part of the world.

The SCD scores (SMCQ) showed a correlation with lower BMI (<18.5), although this was not statistically significant. This suggests that some of older adults population may have experienced weight loss associated with cognitive disorders such as AD. Weight loss has been identified as a marker for the transition from MCI to AD and as a non‐cognitive sign of PCAD.^20^, ^21^ In our population, multiple factors could have contributed to both cognitive difficulties and weight loss, such as poor nutrition, inadequate physical activity, and reduced cognitive stimulation and loss of social connections. Sensory impairment in older adults, a known factor affecting cognitive function,^22^ was not a contributive factor in our study, as individuals with sensory impairment were excluded from the study.

Another surprising finding was that diabetic and hypertensive status did not differ significantly between individuals with and without cognitive impairment. It is well known that both of these conditions are associated with increased risks of all‐cause dementia including Alzheimer's dementia.^23^, ^24^ One possible explanation for this finding in our study is that all participants were regularly followed up by a general practitioner and had routine screening of their blood sugar and blood pressure levels, with many being on medication. Although a cross‐sectional evaluation of blood sugar and blood pressure at the time of the study could have provided more insights into the relationship between vascular and metabolic risk factors and SCD or cognitive function, this was beyond the scope of our research. Given the high prevalence of SCD despite adequate control of these factors, we hypothesize that cognitive difficulties in this population may have other underlying etiologies.

Another intriguing finding was the comparatively lower subjective memory complaints in individuals with objective evidence of cognitive impairment (MoCA score ≤25) compared to those who had cognitive functions within normal range (MoCA score >25). It is quite common that increasing cognitive impairment makes older adults anxious and they underreport the subjective experience and impact of the difficulty, akin to denial in psychological parlance.^25^ Another possibility is that those with cognitive impairment could be actually losing their ability to correctly gauge their impairment, described as anosognosia of one's own cognition.^26^

*Limitations*: Although the sample size was adequate for the problem to be studied, the study did not fully represent all strata of society. The scale used to describe subjective memory complaints may not have captured all those with SCD as defined currently, but it has proved to be a highly sensitive questionnaire valid in the Indian population. Whereas all efforts were made to avoid selection bias, randomization with much larger samples would have made the finding even more robust. Although the MoCA score is adjusted by adding one point for individuals with fewer than 12 years of education, this remains a limitation as it may not fully account for the impact of education on cognitive performance.

*Generalizability (external validity) of the study results*: Overall, our study is the first major community‐based assessment of older adults population specifically focused on SCD, which could be an indicator of PCAD. Unlike previous studies, we extended our investigation to include individuals across all categories of cognitive function, from those with NC (based on standardized screening tools) to those showing decline on objective cognitive tests.

The high prevalence of SCD in this community suggests a potentially increased prevalence of emerging cognitive disorders. Our study also revealed a significant gap in identifying even those with mild to moderate cognitive decline in the community.

The feasibility of using simple, highly sensitive scales to assess SCD in the community, followed by cost‐effective blood biomarkers with high specificity, could provide an affordable strategy for identifying and monitoring elderly individuals at risk of neurodegenerative disorders like AD in LMIC countries. Allocating sufficient resources to integrate this into primary health care or urban health center screening programs, as well as in general practitioner clinics, should be seriously considered by both state and central governments to support the aging population. This approach should be accompanied by scaling up services that provide DMTs for AD to those accurately identified as likely to benefit from them.

## CONFLICT OF INTEREST STATEMENT

Dr Jayakumar Menon has worked as sub‐investigator in a multinational drug trial for schizophrenia by Pfizer. The remaining co‐authors have no conflicts of interest to disclose. Author disclosures are available in the Supporting Information.

## CONSENT STATEMENT

All human subjects provided written informed consent.

## ETHICS STATEMENT

The study was approved by the Institutional Research Ethics Committee of Sri Ramachandra Institute of Higher Education and Research, Chennai, India. The abstract of this article has been published in *Alzheimer's & Dementia*. https://alz‐journals.onlinelibrary.wiley.com/doi/10.1002/alz.079603.

## Supporting information



Supporting Information
